# The Ethical Advertiser

**Published:** 1899-08

**Authors:** F. T. Rogers

**Affiliations:** Providence, R. I.


### THE ETHICAL ADVERTISER.[1]

BY F. T. ROGERS, M.D., PROVIDENCE, R. I.

WERE any excuse needed for this, a plea for the exercise of common sense and the elimination of prejudice in the consideration of our relations as physicians to the public and to the profession at large, it would be found in the fact that not only are the provisions of the code of ethics regarding advertising misunderstood by many and persistently disregarded by more, but there seems to be an impression that the clause of Article I., Section 1, which says that the physician should entertain a due respect for his seniors who have by their labors brought it to the elevated condition in which he finds it, concedes to the older members certain advertising privileges denied to the younger.

That the provisions are misunderstood is evidenced by the following extract from an editorial in an influential medical journal:

"*The Advertising Physician and the Code.*—A physician may advertise with perfect propriety, provided he confines the wording of the advertisement within the limits prescribed by the code of ethics. It is perfectly legitimate for him to put his professional card in the newspapers, this card announcing his name, degree, and address, also his office hours and telephone connections, if he should desire to add these. He may even announce in his card that he is doing special work, or is giving his time and attention to one or more special lines of practice, provided his advertisement reads

---

[1] Read before the American Academy of Dental Science, February 1, 1899.

'practice limited to' these special lines. He cannot say 'special attention given to' any particular class of cases without violating the code."

The American Medical Association has nevertheless distinctly declared that cards in medical journals calling the attention of professional brethren to themselves as specialists are in violation of the code of ethics.

That the provisions are disregarded is a fact known to us all. When the physician occupied a unique position in the community, when the degree of doctor of medicine was synonymous with education and accorded to its possessor all the dignity of a member of a learned profession, when overcrowded ranks with the struggle for, not honor and emoluments, but existence had not aroused the petty jealousies and acrimonious competition which marks our life to-day, the code of ethics represented all that was honorable and worthy of emulation in our professional life, but is, in its entirety, no more adapted to our needs to-day than is the Declaration of Independence to our ultimatum to Spain upon the Cuban question.

The underlying principles are undoubtedly true, but their applicability to the changed conditions of this the close of the nineteenth century, are so susceptible of various interpretation that they might as well not exist.

The incongruities alone displayed by the adherents of the code are enough to invalidate its provisions and in no one thing is this more marked than in the way we advertise our calling and incidentally our own peculiar virtues and qualifications. Yielding to none in admiration of the high calling of our profession, of the blessedness of administering to the ills of mankind, the alleviation of suffering and comfort to the afflicted, it is nevertheless true that we all of us to-day practise medicine for the livelihood it affords us; that we are enabled to do our duty to the State as good citizens, to maintain our families, to educate our children, solely by the remuneration gained by the exercise of our knowledge and skill.

Does any one deny this? Then he looks upon facts through an astigmatism I cannot measure and he and I are not taught in the same school of logic, and argument is uncalled for; but if agreed we are brought face to face with the condition which affronts every student of medicine when with diploma and license in hand he stands at the threshold of life and asks himself, Having complied with the laws of my country, the requirements of my university, the exactions of the code of ethics of my profession, what shall I do to gain a practice and a livelihood?

There was a time when all that was necessary was to announce that he was prepared and ready to practise physic, but that was not A.D. 1899. We allow him in this enlightened age to modestly place his name upon a door-plate, to tell his friends and near neighbors that he is now a practising physician, and, although frowned upon, it is not absolutely forbidden that a short local may appear in the town paper that Dr. A, who recently graduated, etc., is now located in B. Moreover, he is allowed to join the local society, the State society, to call upon the older physicians, but in general he is expected to wait for patients to come to him. Too much energy is not meet in one so young, and his merits must be better recognized before he may indulge in the generous advertising that adds dollars and patients to the coffers of his elders.

This same spirit of forgetfulness, not of self, but of others, is found throughout the game of life and the notoriety achieved by newspaper mention, so entirely proper for those whose reputations are made, is but presumption in the young man and considered unseemly advertising. When a fair measure of success meets his long-continued efforts and he seeks the wider field of specialism he meets the same spirit of opposition. He may practise a specialty,—any one, of course, is at liberty to do that,—but he must not tell any one of it; that is reserved for his elders and betters who have been longer in the field.

Alas! that such a discussion is necessary; that we cannot live in peace and harmony, assured that our merits will be appreciated and that we shall receive due reward for our patient and painstaking study and the years of preparation; but we live in a commercial age teeming with activity, filled with energy, when it is not always the survival of the fittest, but often of the cheekiest, which occurs, and we are, in justice to ourselves, our families, and our children, forced to make some extraordinary effort to increase our business and income,—in short, to advertise, and the question is, how to do it.

Recalling our school-boy days, with their dreaded compositions, we may say, as we did then, "There are many ways of advertising, a few of which I will mention."

One method is beyond cavil. Thorough and conscientious preparation, earnest and undivided attention to our profession, avoidance of unseemly conduct, and honesty and manliness in our relations to the public will advertise our skill, enlarge our reputation, and make our success when nothing else will suffice; and these attributes must be maintained whatever system of advertising we adopt, if we are to succeed, for without them, in the sense in which I employ the word, success is impossible.

Environment should be an important factor in the matter of legitimate advertising. What may be entirely proper in some localities would be manifestly out of place in others. In many of the smaller towns, notably in New England and in the West, it has been the custom of physicians to insert in the local paper and in medical journals a card giving address and office hours. In larger cities this plan is not followed, but it would be a rash assertion to accuse the suburban physician of any great breach of ethics for this action. If it be asserted that such a step would be the entering wedge to the indiscriminate advertising of quackery, we place little reliance upon our own sense of propriety and at once admit our inability to decide for ourselves between right and wrong, relying rather upon some one else to tell us what to do.

The distinction between the professional card in the newspaper and the generous use of editorial or reportorial courtesy in reporting our comings and goings, our patients and operations, as advertisements pure and simple, is hard to define and the advantage is all on the side with the card.

We admit our names, addresses, and office hours to the directory, to the telephone book, and we stamp it on our cards and stationery for the purpose of giving information to the public. Why not go a step farther and allow it to be placed with other professional cards in the local paper for the same reason? Is it any more disreputable than to allow to appear in the daily press such items as "Dr. A, the head surgeon of —— Hospital, last night lectured before the Biological Club upon appendicitis, a subject upon which he is considered an expert;" or, "Mr. A, whose serious illness was chronicled in these columns, is now improving. Dr. B, the eminent surgeon from ———, yesterday performed the successful operation for stone;" or, "Mrs. D, who recently went to ——— to consult the famous oculist, Dr. C, has returned with restored sight, the delicate operation for cataract having been successfully performed by the surgeon." Yet these items have appeared in the daily press without complaint, and Drs. A, B, and C are all members of the American Medical Association.

It is no argument to assert that the first form is a paid advertisement and the latter a spontaneous outburst of generous appreciation. We were not born yesterday, and we know that these articles do not, as a rule, appear without tacit consent and oftentimes direct request.

This one feature of newspaper notoriety is the peculiar property of the ethical advertiser, whether garbed as a news item, an interview on some particular disease or its victims, an ostentatious display of degrees or hospital appointments tacked on to the mere mention of his name, the daily bulletin of the attending physician to an invalided public man, or the more transparent form of the wonderful surgical operation or exhaustive discussion before the medical society,—they are all merely an advertisement, and, as such, are either right or wrong.

If right, then the other simpler forms of advertising are right; if wrong, then the offenders should not be the judges of the enormity of the faults of others.

A national society, to which it is an honor to belong, has a by-law which states that any one who announces in any way that he is engaged in special work is ineligible to membership. For this reason honest, capable men, who have practised a specialty for years, who enjoy the esteem of the profession and the community, yet who have, in any way, announced that their practice was limited to a certain branch, are not eligible, while men, who are already members, violate the rule with impunity.

In such a society, which rejected a candidate because, when he ceased general practice and began special work, he sent a printed letter to his own patients which stated that fact, action has not yet been taken to reprimand the member who allowed a daily paper to photograph him in the performance of an operation and, besides describing his skill as an operator, stated specifically his special practice; nor the other member, who allowed a sketch of his life to appear in a Sunday paper with the statement that in his particular line he was unexcelled in this country, that he frequently performed a certain operation in less than a minute, that he caused no pain, had an extensive practice, saw patients from all over this country, and required two assistants in his office.

The letter of the first offender probably reached a few hundred people, the advertisement of this greater offender reached hundreds of thousands, and doubtless it served its purpose, and brought cases to this surgeon which would otherwise never have known of his existence.

In another society the adoption of such a by-law compelled the withdrawal of two members of years' standing who, living in small towns, had followed the custom of that locality, and had inserted in the local paper a card stating that their practice was limited to certain branches of medicine. The adoption of such a rule did not, however, bar another member from reading a paper before a local society and sending a reprint to thousands of the profession with the containing envelope stamped in large type with the title, the author's name and hospital appointments, which thoroughly advertised his specialty. One is an injustice; the other is ethical advertising.

What would happen to the man who, devoting himself to surgery, should send to every physician in his State a card like this:

"I. Bidwell, M.D., LL.D., Professor of Surgery, ——— Medical College; Surgeon to ——— Hospitals; Consulting Surgeon to ——— Hospitals."

Is that an advertisement? Is it ethical? The American Medical Association has on record that it is not. Then is the prospectus of the medical college with its long list of professors and their qualifications an advertisement? So the post-graduate school, and the hundreds of thousands who receive their catalogues are incidentally reminded that in such a city Professor A is a prominent surgeon and a good man to whom he may refer his patients when needing advice in his line.

To multiply examples of similar methods so familiar to us all, to speak of the man who uses his church affiliation, his society, his personal friends, and his family to advance his own interests, to describe the man who works the politics of his town for his own good, or the man who has always patients who are dangerously ill while those of others would recover anyway; the man who is always busy, who delights in quoting his visiting list, enumerating his cases, mentioning his patients by name, and proffering advice for other than his patients would be an implication of ignorance to this body. Neither need we mention the man who sends his name to the medical society as desirous of reading a paper upon some topic, yet who has no intention of so doing; the man who reads a paper, not to announce his opinion, to excite discussion, or to add to the sum of human knowledge, but simply to advertise himself, his methods, his operation, and to have the same properly quoted in the home paper; the man who judiciously sees that the reporter gets an abstract of his paper for the morrow's issue as one of the principal features of the session; the man who writes a testimonial for pecuniary reasons; the man who tacks his name upon an instrument or operation; nor the man who seizes upon every sort of an object to rehearse his qualifications for the benefit of the public. These are all methods of the ethical advertiser. They are recognized by each of us and we can each recall more than one offender, —doubtless we are somewhat guilty ourselves.

The part a man plays in the drama of life is infinitesimal. The influence of his life is, in most cases, not appreciated. To few of us is granted the power to mould opinion or inaugurate new methods, but the power of a body like the American Academy of Medicine is vastly greater, its seal of approval or disapproval can make or mar, and it is with this idea that I venture to suggest that this subject of advertising is one properly for your consideration.

A recognition of the changed conditions of life, of the mercantile spirit of the age as well as the infractions of the spirit of the code of ethics if not the letter, and a clear statement of what should and should not be considered ethical in this matter, is a task which may well invite your attention.

Gentlemen, this is no wail of a disappointed man, no rebellion against adversity, no socialistic endeavor to controvert existing conditions, but an honest opinion that we need a new chapter to the code of ethics, and that it is the privilege of this body by discussion and action to inaugurate a needed reform. Personal opinion can have but little weight. What I think or what you think is perhaps of little moment, but, nevertheless, I believe that the physician of to-day should have more or less latitude in advertising.

More in a purely medical way, the right to insert a card in medical periodicals, meant only for professional eyes, in the right to assert upon his card and sign that he practises surgery, gynæcology, or ophthalmology, in localities where custom has established a precedent to insert his name and office hours, but not his specialty in the daily press, less latitude in the advertising which reaches the public. The lay press has no part in the life of the physician save to record his obituary. The hundred and one ways of reaching the public through the columns of the daily press should be absolutely tabooed. Reports of the technical proceedings of medical societies should not appear in their columns. Successful operations, curious cases, wonderful cures, as reported in our daily papers, but pamper to a morbid craving and serve no other purpose than to antagonize the best efforts of the profession, to benefit the human race. Quackery thrives by reason of the false opinion gained by the laity from reading just such trash. Restrict our medical opinions save when pertaining to public health and hygiene to medical mediums and let the other and disreputable methods of advertising mark as a quack the one who indulges in or allows it.

